# Rifampin monotherapy for multidrug-resistant *Chryseobacterium indologenes* meningitis unresponsive to trimethoprim-sulfamethoxazole: a case report and review of the literature

**DOI:** 10.3389/fmed.2025.1701719

**Published:** 2026-01-06

**Authors:** Dongchuan Shao, Zhe Li, Zhiwei Cao, Zhou Yang, Wenbiao Huang, Kuairong Pu, Jun Wu, Nan Zhao

**Affiliations:** Department of Neurosurgery, The First Hospital of Kunming (Affiliated Calmette Hospital of Kunming Medical University), Kunming, China

**Keywords:** brain abscess, *Chryseobacterium indologenes*, meningitis, multidrug-resistant, rifampin

## Abstract

Multidrug-resistant (MDR) *Chryseobacterium indologenes* is an emerging pathogen causing challenging central nervous system (CNS) infections, for which there are no standardized treatment guidelines. A systematic review of the literature indicates that such infections are extremely rare. Although trimethoprim-sulfamethoxazole (TMP-SMX) is the most frequently reported first-line therapy, its clinical efficacy is not universal, posing a significant therapeutic challenge. We present the case of a 25-year-old postpartum woman who developed MDR *C. indologenes* meningitis and a brain abscess after undergoing a neurosurgical procedure. The infection did not respond to initial treatment with meropenem or a subsequent course of the first-line agent TMP-SMX. Based on antimicrobial susceptibility testing, therapy was switched to rifampin monotherapy, which led to rapid clinical and microbiological recovery. The patient completed a 24-day course of rifampin and remained recurrence-free during a 20-month follow-up. This is the first report of successful rifampin monotherapy for an MDR *C. indologenes* CNS infection in a patient unresponsive to TMP-SMX. When considered alongside existing literature, these findings highlight the essential role of susceptibility testing and establish rifampin as an important salvage therapy for this life-threatening infection, particularly when recommended first-line treatments fail.

## Introduction

Postoperative central nervous system (CNS) infections represent a formidable clinical challenge in neurosurgery, with infections driven by rare, intrinsically multidrug-resistant (MDR) organisms being a source of particular concern (1, 2). Among these pathogens, *Chryseobacterium indologenes* (*C. indologenes*) has emerged as an especially troubling nosocomial opportunistic pathogen (3). First isolated in 1993 from the tracheal aspirate of a patient with ventilator-associated pneumonia, this Gram-negative bacillus is deceptively widespread in natural environments yet possesses a remarkable capacity for nosocomial colonization—thriving in water systems, resisting chlorination, and forming protective biofilms on medical equipment (4, 5). Worryingly, its clinical incidence has reportedly increased in tandem with the widespread use of last-resort antibiotics such as polymyxins and tigecycline (6).

The clinical management of *C. indologenes* is exceptionally complicated by its intrinsic multidrug resistance, which is primarily driven by its ability to co-produce both class A extended-spectrum β-lactamases and class B metallo-β-lactamases (MBLs), conferring natural resistance to multiple antibiotics including carbapenems (7). Infections typically occur in vulnerable patients with well-defined risk factors, such as immunocompromised states, prolonged hospitalization, and the presence of indwelling medical devices. While most often causing bacteremia or respiratory infections, its rarest and most dangerous manifestation is in the CNS, a complication almost exclusively linked to neurosurgical procedures (8, 9).

Although reported globally, cases demonstrate a notable geographic concentration, with the majority of reports originating from Taiwan, China. However, due to the overall scarcity of cases, the lack of global data has precluded the formation of standardized treatment guidelines for *C. indologenes* CNS infections. This leaves clinicians to navigate therapeutic decisions based on a small collection of isolated case reports.

To address this critical gap, we herein report a challenging case of refractory meningitis and brain abscess caused by MDR *C. indologenes*. Concurrently, we provide a systematic review and summary of the existing literature to offer a comprehensive analysis of this rare clinical entity and to provide a valuable reference for clinicians facing this formidable pathogen.

**Figure 1:**
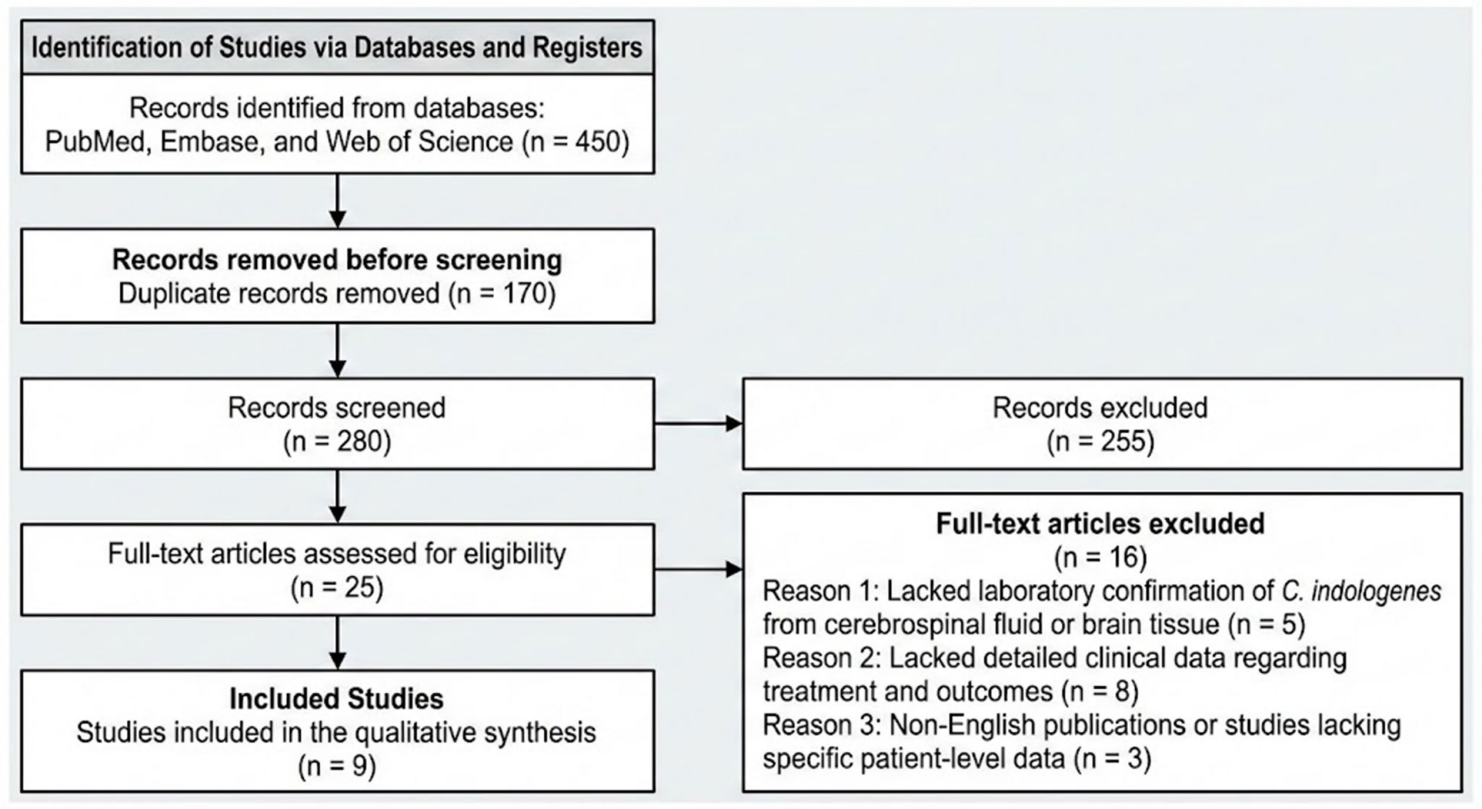
PRISMA 2020 flow diagram of the literature search and selection process. This flowchart details the systematic screening strategy applied across the PubMed, Embase, and Web of Science databases. It illustrates the progression from initial record identification to final inclusion, specifying the number of records excluded at each stage and the reasons for exclusion during the full-text assessment (e.g., lack of laboratory confirmation or insufficient clinical data). Ultimately, nine studies were identified as eligible for qualitative synthesis.

A comprehensive literature search was conducted in PubMed, Embase, and Web of Science databases to identify reported cases of *C. indologenes* central nervous system infections published from the database inception up to August 2025. The search strategy employed a combination of Medical Subject Headings (MeSH) and free-text terms, including “*Chryseobacterium indologenes*” OR “*Flavobacterium indologenes*” combined with “meningitis,” “brain abscess,” “ventriculitis,” or “central nervous system infection.” To ensure a rigorous selection process consistent with the PRISMA (Preferred Reporting Items for Systematic Reviews and Meta-Analyses) guidelines (Figure 1), two independent reviewers screened the titles and abstracts. Inclusion criteria were defined as: (1) case reports or case series involving human subjects; (2) laboratory-confirmed *C. indologenes* infection from cerebrospinal fluid or brain tissue; and (3) availability of detailed clinical data regarding treatment and outcomes. We excluded duplicate records, non-English publications, and studies lacking specific patient-level data. This systematic screening process yielded a total of nine relevant cases, which were subsequently analyzed alongside the present case to characterize the epidemiological and therapeutic patterns of this rare infection.

The systematic screening process identified nine relevant cases of *C. indologenes* CNS infections suitable for qualitative synthesis (Table 1). An analysis of these cases, combined with the present report, reveals a bimodal age distribution, predominantly affecting infants (often with prematurity or congenital hydrocephalus) and adults with history of neurosurgical interventions. The majority of reported cases involved device-associated infections, particularly ventriculoperitoneal or lumboperitoneal shunts. In terms of antimicrobial management, trimethoprim-sulfamethoxazole (TMP-SMX) was the most frequently employed agent, often used in combination with fluoroquinolones. Clinical outcomes were generally favorable, with 8 out of 10 patients (including the present case) achieving recovery; however, therapeutic failure with first-line agents remains a critical concern, necessitating alternative strategies for multidrug-resistant strains.

**Table 1:** Clinical characteristics, treatments, and outcomes of previously reported cases of *C. indologenes* central nervous system infections.

Author (year)	Age/Gender	Underlying conditions	Invasive procedures	Prior antibiotic exposure	None identified	Antimicrobial therapy administered	Outcome
Al-Tatari (36)	13 years/M	Hydrocephalus, intracranial infection (MSSA)	Lumboperitonial (LP) shunt	Vancomycin	Ciprofloxacin piperacillin/tazobactam, rifampin, TMP-SMX	TMP-SMX rifampin IV 14 days	Recovered
Ceylan (37)	2 months/M	Hydrocephaly	External shunt	Ampicillin-sulbactam, cefotaxime	Ampicillin-sulbactam, levofloxacin, TMP-SMX	Ampicillin-sulbactam, levofloxacin	Died
Ozcan (38)	6 months/M	Hydrocephaly, prematurity	VP shunt	Vancomycin meropenem	Ciprofloxacin, levofloxacin, cefoperazone, TMP-SMX	(Cefoperazone-sulbactam + TMP-SMX) 14 days	Recovered
Eshwara (39)	6 days/F	Sepsis	External shunt	Piperacillin-tazobactam amikacin	Ciprofloxacin, TMP-SMX	Ciprofloxacin (30 mg/kg/day IV 4 weeks), ciprofloxaci + TMP-SMX (orally 2 weeks)	Recovered
Hendaus (40)	8 days/F				Cefepime TMP-SMX	Cefepime IV 3 weeks	Recovered
Olbrich (41)	11 months/M	CSF leakage hydrocephalus	Ventriculostomy		Ceftazidime, ciprofloxacin, TMP-SMX	TMP-SMZ (8 mg/kg/12 h IV TMP) ceftazidime (50 mg/kg/6 h, IV) 21 days	Recovered
Wang (42)	75 years/M	Intracranial, aneurysm pneumonia	Aneurysm clipping + LED	Teicoplanin	TMP-SMX	TMP-SMX (0.4 g IV Q12) + SMX (0.96 g orally Q12H)	Recovered
Sud (43)	28 years/F	Medulloblastoma	EVD		Minocycline, TMP-SMX	TMP (80 mg)/SMX (0.4 g) orally bid 7 days	Recovered
2021 our case	26 years/F	Hydrocephalus encephalopyosis	LED LCPS, LP	Meropenem	No discover	Rifampin	Recovered

EVD, external ventricular drainage; LP, lumbar puncture; LP shunt, lumboperitoneal shunt; MSSA, methicillin-susceptible *Staphylococcus aureus*; TMP-SMX, trimethoprim-sulfamethoxazole; VP, ventriculoperitoneal.

## Case description

**Figure 2:**
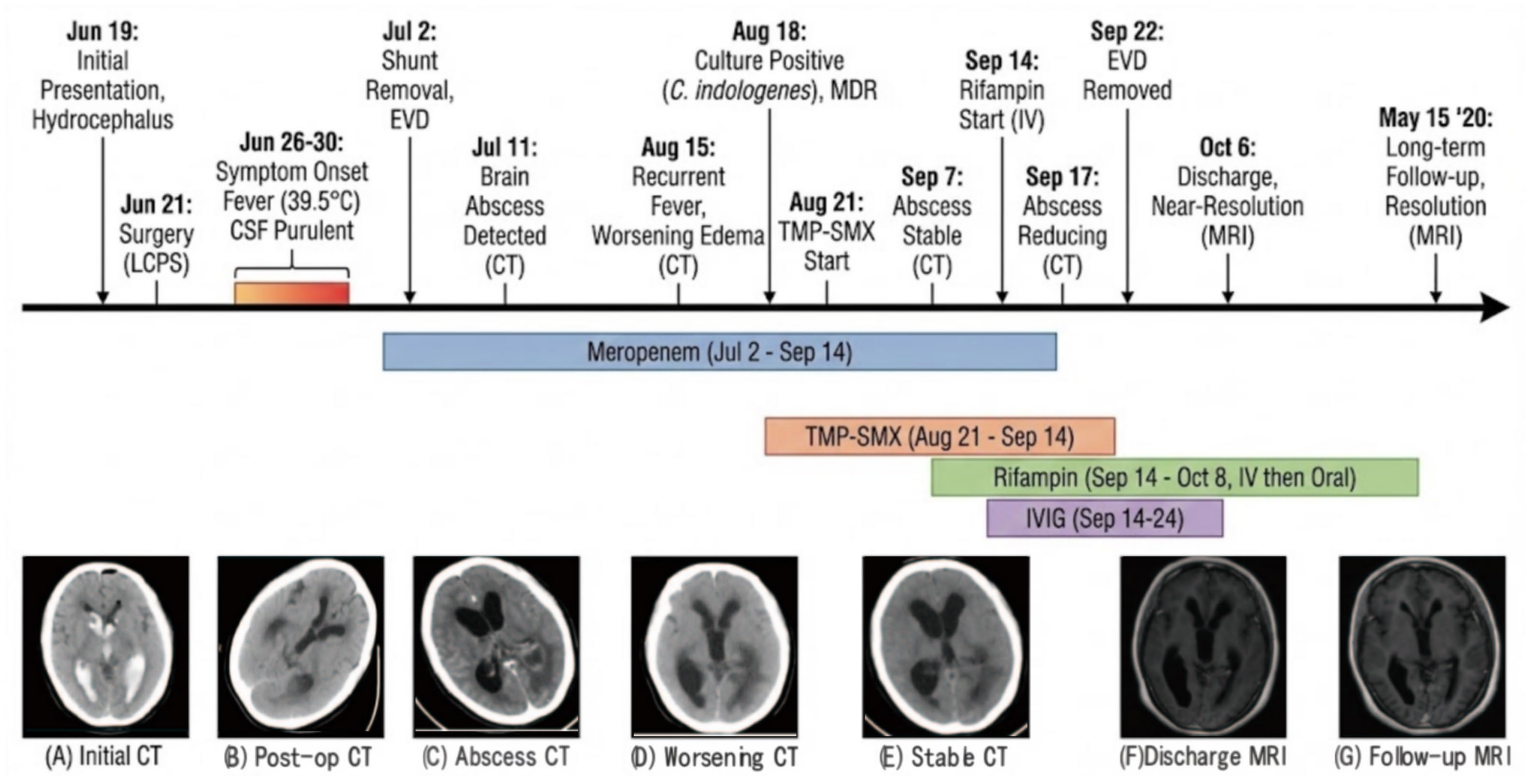
Comprehensive timeline of clinical course and serial neuroimaging. The upper panel illustrates the chronological sequence of major clinical events, surgical procedures (EVD, external ventricular drainage; LCPS, lumboperitoneal shunt), and microbiological findings. The colored bars represent the duration of antimicrobial regimens: blue (meropenem), orange [trimethoprim-sulfamethoxazole (TMP-SMX)], and green (rifampin), along with adjunctive intravenous immunoglobulin (IVIG, purple). The lower panel displays representative neuroimaging scans obtained at key time points: **(A)** June 19, initial CT scan showing intraventricular hemorrhage; **(B)** June 22, postoperative CT demonstrating improved hydrocephalus following LCPS; **(C)** July 11, contrast-enhanced CT revealing the formation of a brain abscess; **(D)** August 15, CT scan showing worsening perilesional edema and abscess recurrence; **(E)** September 7, CT scan showing stable abscess size following TMP-SMX therapy; **(F)** October 6, contrast-enhanced MRI at discharge showing near-complete resolution; and **(G)** May 15, 2020, long-term follow-up MRI demonstrating sustained resolution without recurrence.

A 25-year-old woman, 33 days after cesarean section, was admitted to our hospital on June 19, 2018, for the management of communicating hydrocephalus, presenting with a five-day history of headache and vomiting. Her significant medical history included moyamoya disease, which had caused an intraventricular hemorrhage 1 month prior (at 36 weeks of gestation); this was managed at our institution with bilateral external ventricular drainage (EVD) (Figure 2) and a subsequent cesarean section. The patient had no family history of cerebrovascular diseases, genetic disorders, or psychosocial issues. Upon the current admission, an initial evaluation including cerebrospinal fluid (CSF) analysis and a contrast-enhanced head CT revealed no signs of intracranial infection (Figure 2). The patient’s overall clinical course is summarized in Figure 2.

**Figure 3:**
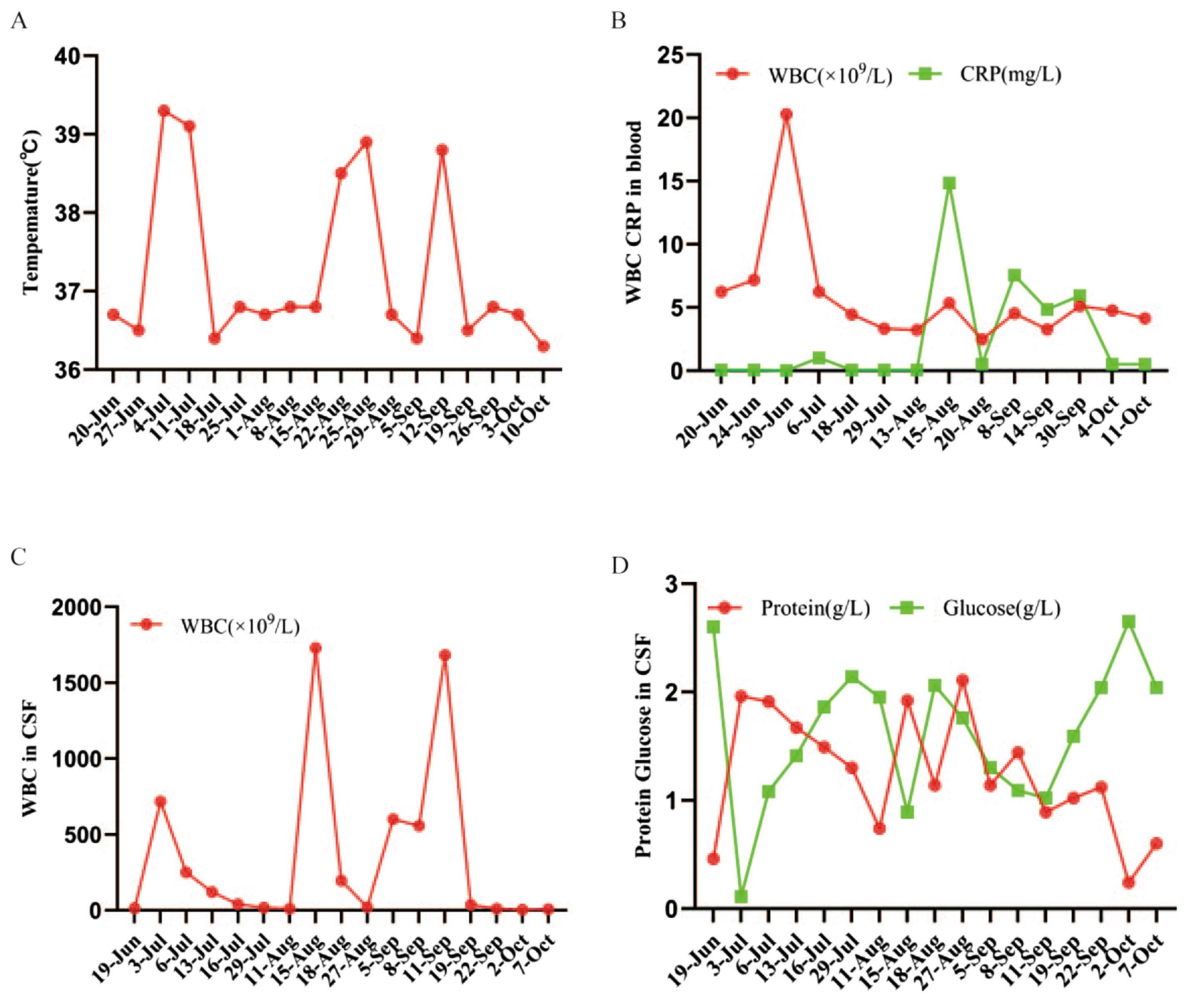
Dynamic changes in clinical and laboratory parameters over the course of treatment. **(A)** Changes in systemic inflammatory markers in the serum, including the white blood cell (WBC) count and C-reactive protein (CRP) level. These markers peaked during the acute phase of the infection and subsequently declined with effective treatment. **(B,C)** Changes in cerebrospinal fluid (CSF) parameters, including WBC, protein, and glucose levels. After the therapy was switched to rifampin, a marked decrease in CSF pleocytosis was observed, and the biochemical markers normalized. **(D)** Changes in the patient’s daily maximum body temperature. A rapid and sustained defervescence was observed following the initiation of rifampin.

The patient underwent a lumboperitoneal shunt placement under general anesthesia on June 21, 2018. Although her initial postoperative recovery was good with improved hydrocephalus on a follow-up CT (Figure 2), she developed a headache on June 26 (Figure 2) and a high-grade fever of 39.5 °C by June 30. Laboratory findings revealed a significant inflammatory response, including leukocytosis (WBC count 20.28 × 10^9^/L) and an elevated C-reactive protein level (7.8 mg/L) (Figure 3). CSF analysis showed typical purulent changes: a WBC count of 4,914 × 10^6^/L (93% polymorphonuclear cells), elevated protein (1.7 g/L), and decreased glucose (0.08 g/L) (Figure 3).

Based on the diagnosis of intracranial infection, empirical therapy with intravenous meropenem (1 g every 8 h) was initiated, and on July 2, the peritoneal end of the shunt was removed and converted to an external lumbar drain. Despite multiple initial CSF cultures being negative, a contrast-enhanced CT ultimately revealed a left ventricular trigone abscess (Figure 2). Under meropenem, the abscess showed an initial reduction in size (Figure 2), but the patient’s condition deteriorated again on August 15 with a recurrent high-grade fever (Figure 3) and worsening perilesional edema on CT (Figure 2).

The pivotal breakthrough occurred on August 18, when a CSF culture finally identified the causative pathogen as *Chryseobacterium indologenes*. The cerebrospinal fluid samples were inoculated onto 5% sheep blood agar and chocolate agar plates and incubated at 35 °C in a 5% CO_2_ atmosphere for 24 h. Bacterial identification was confirmed using matrix-assisted laser desorption ionization-time of flight mass spectrometry (MALDI-TOF MS) (Bruker Daltonics, Germany) Routine antimicrobial susceptibility testing was performed using an automated VITEK 2 Compact system (bioMérieux, France), which provided minimum inhibitory concentration (MIC) values for the antibiotic panel (Table 2). However, as rifampin susceptibility testing for *C. indologenes* was not available on the automated panel, it was additionally assessed using the Kirby–Bauer disk diffusion method on Mueller–Hinton agar, strictly adhering to the Clinical and Laboratory Standards Institute (CLSI) M100 guidelines (0.5 McFarland standard inoculum, 5 μg rifampin disk). The treatment was subsequently adjusted to oral trimethoprim-sulfamethoxazole (0.96 g, twice daily). Despite marked radiographic improvement of the abscess (Figure 2), the patient’s fever did not resolve (Figure 3), and subsequent CSF cultures remained positive, indicating that the second treatment regimen had also failed (Figure 2).

**Table 2:** Antimicrobial susceptibility profile of the *C. indologenes* isolate from cerebrospinal fluid (CSF).

S. No.	Antibiotics	Minimum inhibitory concentration value (μg/mL)	KB (mm)	Interpretation
1	Rifampin		24	Unidentifiable
2	Piperacillin/Tazobactam	≧128		Resistant
3	Imipenem	≧16		Resistant
4	Meropenem	≧16		Resistant
5	Amikacin	≧64		Resistant
6	Gentamicin	≧16		Resistant
7	Ciprofloxacin	≧4		Resistant
8	Aztreonam	≧64		Resistant
9	Tigecycline	≧8		Resistant
10	Tobramycin	≧16		Resistant
11	Ceftriaxone	≧64		Resistant
12	Levofloxacin	≧8		Resistant
13	Macrodantin	≧512		Resistant
14	Ampicillin	≧32		Resistant
15	Tetracycline	≧16		Resistant
16	Cefepime	≧64		Resistant
17	Amoxicillin/clavulanic acid	≧32		Resistant
18	TMP-SMX	≧320		Resistant
19	Ceftazidime	≧64		Resistant
20	Cefazolin	≧64		Resistant
21	Cefoxitin	≧64		Resistant

The Kirby–Bauer disk diffusion test for rifampin yielded a zone of inhibition of 24 mm. A formal interpretation (i.e., susceptible, intermediate, or resistant) could not be determined, as the Clinical and Laboratory Standards Institute (CLSI) has not established interpretive breakpoints for *C. indologenes*.

Given the failure of the first two regimens, all antibiotics were discontinued on September 14, and therapy was switched to intravenous rifampin (0.6 g, once daily). The clinical response was dramatic, with the patient’s fever resolving within 24 h (Figure 3). Following a 10-day intravenous course, she was transitioned to a 14-day oral regimen of rifampin (0.45 g, three times daily), with adjunctive intravenous human immunoglobulin. This was accompanied by a rapid normalization of systemic inflammatory markers (Figure 3) and CSF parameters (Figure 3). Radiological imaging confirmed progressive improvement and eventual resolution of the abscess and hydrocephalus (Figure 2). On September 22, 2018, after confirming that CSF cultures were persistently negative, the external drain was successfully removed.

The patient was discharged 2 weeks later after making a full recovery. On long-term follow-up, an MRI of the head performed on May 15, 2020—nearly 2 years post-procedure—showed no signs of recurrence of the infection or hydrocephalus (Figure 2), and she has remained asymptomatic.

The patient expressed great relief and gratitude upon her recovery, particularly noting the resolution of her severe headache and fever which had significantly impacted her ability to care for her newborn. She reported no long-term neurological deficits during the follow-up visits and was satisfied with the explanation regarding the complexity of her infection and the rationale for the multiple changes in antibiotic therapy.

## Discussion

Postoperative CNS infections remain a formidable challenge in neurosurgery, associated with significant morbidity and mortality (10). This challenge has been amplified over the past decade by a rising proportion of infections caused by Gram-negative bacilli, which have increased from 12 to 27% (11, 12). It is within this context of evolving epidemiology that rare opportunistic pathogens like *C. indologenes* have become a source of growing concern (13). Despite an increase in its overall isolation rate, linked to broad-spectrum antibiotic use, CNS infection by this organism is exceptionally rare, with only nine cases previously documented in the literature (14). Given the patient’s history of prolonged hospitalization and multiple indwelling devices, the infection was determined to be nosocomial in origin, likely stemming from colonization of the external ventricular drainage or lumboperitoneal shunt catheters.

A systematic analysis of these published cases reveals a distinct bimodal age distribution for *C. indologenes* CNS infections, primarily affecting two vulnerable populations: infants with immature immune systems and adults with immune deficiencies undergoing invasive procedures. Our patient aligns perfectly with the latter group, exhibiting a cluster of well-established risk factors: (1) multiple invasive procedures (ventricular drainage, cesarean section, lumboperitoneal shunt); (2) a state of perinatal immunosuppression; and (3) prolonged exposure to carbapenem therapy.

The therapeutic management of *C. indologenes* is notoriously difficult. Conventional empirical antibiotics for CNS infections, such as vancomycin, linezolid, and carbapenems, are generally ineffective, a finding supported by the SENTRY Antimicrobial Surveillance Program (1997–2001) (15, 16). The organism’s formidable multidrug resistance is primarily mediated by metallo-β-lactamase production, which contributes to a mortality rate as high as 25% as reported by Lin. This challenge is further compounded by the recent emergence of pan-drug-resistant strains, as described by Agarwal’s (17, 18).

Current evidence suggests that the most effective agents against *C. indologenes* are trimethoprim-sulfamethoxazole (TMP-SMX) and certain fluoroquinolones (e.g., gatifloxacin and levofloxacin), with reported susceptibility rates exceeding 95% (19, 20). Other agents like ciprofloxacin, cefepime, piperacillin, and rifampin have also shown significant activity (21). Consequently, Wang et al. have recommended TMP-SMX as the first-line treatment for CNS infections (22). However, the efficacy of TMP-SMX can be precarious; Wang also reported a case of an elderly patient post-intracranial aneurysm clipping who, although initially susceptible, developed resistance after 8 days of therapy (23). The decision to employ rifampin monotherapy warrants careful consideration regarding its pharmacokinetic properties and the potential for resistance (24, 25). Rifampin is highly lipophilic, a characteristic that facilitates its penetration across the blood–brain barrier, particularly in the presence of meningeal inflammation where cerebrospinal fluid (CSF) concentrations can reach therapeutic levels for susceptible organisms (26, 27). Although rifampin penetration is generally considered variable, large-scale surveillance data, such as the SENTRY program, indicate that *C. indologenes* isolates often retain high susceptibility to rifampin, with susceptibility rates reported as high as 85.7% (28, 29). However, the rapid emergence of resistance via point mutations in the rpoB gene (encoding the beta-subunit of RNA polymerase) is a well-documented risk associated with rifampin monotherapy in other bacterial infections (30, 31). In our case, the sustained clinical cure suggests that the specific strain was highly susceptible and that the preceding surgical drainage may have sufficiently reduced the bacterial load to prevent the selection of resistant mutants. Nevertheless, given these risks, we emphasize that rifampin monotherapy should be regarded as a salvage therapy for refractory cases where first-line agents like TMP-SMX have failed, rather than a routine initial recommendation. The unique value of our case lies in being the first to demonstrate the clinical efficacy of rifampin monotherapy against a strain with primary non-response to TMP-SMX (32). This finding is supported by prior literature: Al-Tatari et al. previously reported successful treatment with a TMP-SMX and rifampin combination, and a large analysis by Singh et al. showed a 75% susceptibility rate to rifampin, suggesting it is a viable alternative (33, 34).

Beyond antimicrobial selection, comprehensive management strategies were crucial. For device-related CNS infections, the decision on catheter removal is controversial. While rapid removal is advised for bacteremia, we argue that for patients with shunt-dependent hydrocephalus, a more conservative approach is warranted. Our strategy of converting the lumboperitoneal shunt to an external lumbar drain maintained necessary CSF diversion while allowing for continuous drainage, which was instrumental in controlling the infection. Furthermore, adjunctive intravenous immunoglobulin was administered, consistent with the recognized importance of immunomodulation in severe infections. This case report strictly follows the CARE Guidelines for consensus-based clinical case reporting (35).

## Conclusion

In conclusion, we present the first case of multidrug-resistant *C. indologenes* meningitis with a brain abscess to be successfully treated with rifampin monotherapy, notably after the failure of the recommended first-line agent, trimethoprim-sulfamethoxazole. This experience underscores several critical points: first, the indispensability of antimicrobial susceptibility testing to guide therapy for this rare pathogen; and second, the potential of rifampin as a key salvage agent for refractory cases. While prevention through strict aseptic techniques remains paramount, the successful outcome was ultimately achieved through a comprehensive and individualized management strategy that included precise antimicrobial selection, strategic device management, and host immune support. This highlights the necessity of a multifaceted approach for these life-threatening infections.

## Data Availability

The original contributions presented in the study are included in the article/supplementary material, further inquiries can be directed to the corresponding author.
